# Prevalence and Comorbidities of Anemia in Hospitalized Adults

**DOI:** 10.7759/cureus.79568

**Published:** 2025-02-24

**Authors:** Ahmed A Mohamed

**Affiliations:** 1 Internal Medicine, Thumbay University Hospital, Ajman, ARE

**Keywords:** anemia in hospitalized patients, chronic disease-related anemia, comorbidities in anemia, hemoglobin levels, hospitalized patients, s: anemia

## Abstract

Background: Anemia, characterized by a decline in hemoglobin levels insufficient to meet the body's physiological demands, poses significant health risks. Its unchecked presence in patients has been medically linked to a surge in mortality and morbidity rates and reduced treatment effectiveness.

Objectives: This research aims to (1) determine the prevalence of anemia occurrence among adult patients hospitalized at Thumbay University Hospital, (2) highlight the comorbidities accompanying anemic patients, and (3) assess anemia patterns across different age groups, with a focus on young adults.

Methods: This hospital-centric, observational research analyzed the hematological test results of 437 patients. Participants were classified based on age, gender, hemoglobin concentration at admission, mean corpuscular volume, hospital stay duration, existing conditions and comorbidities at admission, and long-term medications. Anemia was diagnosed if hemoglobin levels were less than 120 g/L in females and under 130 g/L in males.

Results: Among the hospitalized patients, 52.17% exhibited anemia, with females showing a higher prevalence than males. The highest occurrence of anemia was notably among the elderly population, particularly those over 65, while the lowest was found in adults aged between 31 and 40. The majority of cases were attributed to chronic disease-related anemia and infection, with the former being the most common underlying cause.

Conclusion: This research provides an assessment of anemia prevalence in a clinical setting, analyzing a substantial cohort of hospitalized adults. The results reveal a high occurrence of anemia, primarily mild or moderate, among patients admitted to internal medicine and long-term care wards. The study's findings can underscore the frequency of anemia in hospital settings. Further exploration is required to understand the heightened prevalence of anemia among young adults.

## Introduction

Anemia is characterized by a decrease in hemoglobin (Hb) levels to the point where they are insufficient to cater to the physiological needs of the body [1]. It is recognized within medical circles that untreated anemia independently raises the risk of elevated mortality, morbidity, and reduced treatment effectiveness [2]. Despite this, anemia is often overlooked in clinical settings, particularly when other pathologies coexist [3].

Although this study was conducted from May 2020 to August 2021, anemia continues to be a significant concern in hospitalized patients. Recent studies (2022-2023) continue to highlight the role of anemia in increasing morbidity and reducing treatment effectiveness in similar populations, particularly among the elderly and patients with chronic diseases. Therefore, this research remains relevant, offering insights that continue to inform anemia management strategies in clinical settings.

Iron deficiency anemia (IDA) is the most common cause of anemia, frequently observed in hospitalized patients with concurrent underlying health conditions [4]. Additionally, anemia has been associated with an increased frequency of hospitalizations and a longer duration of stay once hospitalized [5].

## Materials and methods

This single-center, retrospective observational study evaluates all adult patients admitted to the internal medicine acute ward, ICU, and long-term care ward over 18 months (May 2020 to August 2021). The study was approved by the Ethical Committee of Gulf Medical University and Thumbay University Hospital in Ajman, UAE.

A review of the hospital electronic database for all internal medicine admissions for adult patients (18+ years of age of both genders, excluding pregnant females) was conducted. The relevant clinical data (age, sex, Hb concentration upon admission, mean corpuscular volume (MCV), length of hospital stay, underlying conditions upon admission, co-morbidities, chronic medications) of each patient was collected in an ad hoc anonymous database. IBM SPSS Statistics for Windows, Version 26 (Released 2019; IBM Corp., Armonk, New York) was employed for categorization and descriptive data analysis.

A total of 437 patients were included in the study; among them, there were 292 males and 145 females. Patients were stratified based on age (18-30, 31-40, 41-50, 51-65, >65 years), on anemia severity in agreement with WHO criteria (mild with Hb 110-129 g/L in men and Hb 110-119 g/L in women, moderate with Hb 80-109 g/L in both genders, and severe Hb <80 g/L in both genders) [1]. Patients were also stratified based on length of hospital stay (>14 days of inpatient care is considered a long-term patient) and the etiology of anemia. The MCV was recorded for each patient and categorized as microcytic (<80 fL), normocytic (80-100 fL), or macrocytic (>100 fL).

Each patient was assessed according to their medical background, laboratory investigations, radiological images and reports, and medical procedures (when deemed appropriate). Then, the responsible causes of their decreased Hb were allocated into the following groups: anemia of chronic disease, infection, gastrointestinal (GI) bleeding, nutritional deficiencies, other types of bleeding, hemoglobinopathies, malignancy, and unknown cause (idiopathic). Multifactorial anemia was used to describe patients with more than one identifiable cause of anemia.

## Results

In general, the prevalence of anemia in our cross-sectional study was 52.17%, with minor differences between the sexes (146 patients, 50% male vs. 82 patients, 57% female), as shown in Table 1. In contrast to the severity of anemia, anemic male patients mainly exhibited mild anemia (42.65% mild, 37.77% moderate, 19.58% severe), while female patients mostly had moderate anemia (34.18% mild, 53.17% moderate, 12.65% severe). According to the MCV value, 57.84% were normocytic, 41.71% were microcytic, and 0.45% were macrocytic.

**Table 1:** Prevalence of anemic patients The prevalence of anemia was calculated based on three key variables: gender, age, and length of hospital stay (LOS). LOS was categorized into short-term (less than 14 days) and long-term (14 days or more).

Variables	Total Patients	Anemic	Prevalence
Total	437	228	52.17%
Gender			
Male	292	146	50%
Female	145	82	56.55%
Age group			
18-30 years	57	35	61.40%
31-40 years	90	43	47.77%
41-50 years	109	38	34.86%
51-65 years	121	65	53.72%
>65 years	60	47	78.33%
Length of stay			
Short-term stay	365	177	48.5%
Long-term stay	72	56	77.78%

The causes of anemia identified in the study in chronological order were found to be as follows: anemia of chronic disease (ACD) (36.47%), infection (30.94%), GI bleeding (9.95%), nutritional deficiencies (9.39%), other types of bleeding (4.98%), hemoglobinopathies (3.31%), idiopathic (2.76%), and malignancy (2.2%). Further categorization of patients according to the number of identifiable causes of anemia each showed that 79.38% had a single identifiable reason. In comparison, 20.62% accounted for more than one cause and were labeled as multifactorial.

Specific analysis was carried out to identify comorbidities related to the anemic patients, as described in Table 2. The most reoccurring conditions were primary hypertension (found in 114 patients), diabetes mellitus (DM) type 2 (85 patients), polypharmacy (59 patients), hyperlipidemia (35 patients), and tracheostomy status (28 patients). These comorbidities were the leading comorbidities in both short- and long-term hospitalizations, with an additional prevalence observed in long-term hospitalized patients suffering from COVID pneumonia and post-COVID status, affecting 13.11% of long-term anemic patients.

**Table 2:** Outlining the most frequently reoccurring comorbidities among anemic patients within our study population

Comorbidity	Total	Anemic
Primary hypertension	210	114 (36.16%)
Diabetes mellitus	154	85 (26.2%)
Polypharmacy	86	59 (17.34%)
Hyperlipidemia	61	35 (12.92%)
Tracheostomy status	31	28 (7.38%)

Due to the COVID-19 pandemic, the study involved a limited cohort of hospitalized COVID patients. These included 19 cases of acute COVID-19 infections (17 were anemic, 89%) and 21 cases of post-COVID status (13 were anemic, 61.9%). Furthermore, out of the 86 patients associated with polypharmacy, 59 were anemic (68.6%).

## Discussion

This observational study aimed to determine the prevalence of anemia, its causes, and associated comorbidities in hospitalized patients. The results indicate that most patients suffer from anemia upon admission, primarily attributable to ACD and infection, as corroborated by numerous prior studies [3,6,7].

Our single hospital study observations illustrated that approximately 50% of the admitted patients had anemia. Regarding the difference between the two genders, most females had anemia, which represented a higher incidence than males. Coincidentally, regional research from neighboring Gulf countries (Saudi Arabia and Oman) has found a similarly high incidence of anemia in females [8,9]. In contrast, a single-center study in Bahrain has found a higher incidence in males [10]. The former suggested a higher incidence in females in the Gulf region and UAE, which is not absolute and can vary depending on various factors.

More than 75% of our patients exhibited mild or moderate anemia, with males predominantly experiencing mild anemia and females, particularly, presenting with moderate anemia. This aligns with findings from numerous studies and reinforces the notion that females are more predisposed to decreased Hb levels to a moderate level during hospitalization [5,11]. On the other hand, severe anemia was similar to mild anemia in that its occurrence was more prevalent in males [3]. Our data reveal that most patients had normocytic anemia, while over a third had microcytic anemia, which fit their clinical presentations and medical conditions.

The findings show a strong correlation between the presence and severity of anemia and prolonged hospital stays. Specifically, the higher percentage of anemic patients with longer hospital stays compared to those with shorter stays suggests an increased risk of developing or worsening anemia over time. This observation aligns with the findings reported in studies [5,6,12,13].

Over three-quarters of geriatric (over 65 years of age) patients in our study had anemia, making them the age group with the highest prevalence. Interestingly, only one-third of the patients aged 41-50 had anemia, while over half of the patients aged 51-65 were prevalent with anemia. This implies an increased risk of anemia after 50 years [10,11,14]. Following the elderly community, the second most prevalent age group was the young adults (18-30 years old). Almost two-thirds of their population had anemia, which was expressed in another study with a similar finding [7]. Still, there is a lack of literature concerning this age group in the context of anemia in hospitalization.

One-fifth of our study population was diagnosed with multifactorial anemia, which is mainly attributed to IDA combined with ACD or infection. In this population subsection, the most relevant chronic conditions were heart failure and chronic kidney failure. Our percentage of multifactorial anemia lies pretty low on the spectrum compared to other studies [6,10], but that can be attributed to the large population of younger adults involved in the study, as multifactorial anemia is most common in older age.

The most common cause of anemia in our study was isolated ACD, found in over a third of the study population, as it was the only known contributing factor to the decreased Hb level in those patients. Surprisingly, the number of isolated IDA cases was less than 10%, which was expected to be higher. A study by Joosten and Lioen on elderly patients presented findings close to ours; their results included 23 patients diagnosed with "IDA only," while "ACD only" was diagnosed in 104 patients [15].

In our research, infection emerges as the second primary cause of anemia, especially chronic infections. Anemia can rapidly manifest in cases following bacterial infections or conditions related to severe sepsis. However, the anemia stemming from chronic disorders is typically not severe or progressive [16]. In addition, GI bleeding is a frequent cause of anemia and a contributing factor to the onset of IDA [17]. In our study, GI bleeding was responsible for two-thirds of anemia due to blood loss. This emphasizes the necessity for proper medical management and treatment of such instances, as they are often associated with a high incidence of malignancy. This is predominantly observed in cases of right-sided colon carcinoma in men over 50 and post-menopausal women with asymptomatic IDA [18].

Anemia represents the most common blood-related complication of cancer, impacting 40-64% of patients undergoing treatment for malignancies [19]. Conversely, hemoglobinopathies rank as the most prevalent single-gene disorders globally, exhibiting significant occurrence in certain regions, notably the Mediterranean and Middle Eastern countries [20]. In our hospital, the cases of malignancies and hemoglobinopathies were limited due to the absence of specialized teams and experts in these respective fields.

As indicated by our study and others [21,22], hypertension and DM type 2 are two of the most common comorbidities associated with anemia in adults. Another reoccurring comorbidity found in 35 patients in our study is hyperlipidemia, which multiple studies [23,24] suggested that IDA may be associated with increased cholesterol levels. Polypharmacy appeared as one of the significant comorbidities, especially in the elderly and patients with chronic kidney diseases. Other studies confirmed the effects of polypharmacy [25-27].

Exclusively in long-term and intensive care patients, a high percentage of prolonged mechanically ventilated patients showed anemia, as per the results of our study and others [28,29]. Furthermore, within this group of patients, a high number of cases displayed an association between COVID pneumonia and anemia, especially in severe and critical COVID-19 cases [30].

This observational study comes with several constraints that should be acknowledged. Firstly, Hb levels were only recorded upon admission or within the first 24 hours of hospitalization. The absence of Hb level tracking throughout the hospital stay restricts the ability of this study to assess the impact of comorbidities on the progression or intensity of anemia. Additionally, the severity of illness at presentation, which can significantly influence anemia status, was not systematically accounted for in our analysis due to the retrospective nature of the study. Future research incorporating standardized severity indices, such as APACHE II or SOFA scores, would help better delineate these relationships.

Secondly, due to a dearth of literature focusing on younger adults, most existing studies primarily target middle-aged adults and the elderly. This indicates that the findings may be more applicable to patients over 50 years of age, although our study highlights the need for further investigation into anemia prevalence among younger hospitalized adults. Lastly, as a single-center study, our findings may not fully represent anemia prevalence across all hospitalized patients in the UAE. The retrospective design further limits our ability to establish causal relationships, as data collection was constrained to available medical records.

## Conclusions

This study provides valuable data on the prevalence and comorbidities of anemia in hospitalized adults, highlighting its significant impact in clinical settings. Anemia was most prevalent in patients over 65, with a notable occurrence in adults under 30, primarily presenting as normocytic anemia linked to chronic diseases. The increased prevalence in middle-aged individuals, peaking in the sixth decade, underscores the necessity of integrating routine anemia screening and age-specific management strategies into hospital protocols. These strategies should include tailored interventions for different age groups, particularly focusing on the elderly and middle-aged patients, while addressing the unique risk factors for younger adults. Additionally, the unexpectedly high rates of anemia in younger adults emphasize the need to investigate specific risk factors in this group, such as nutritional deficiencies or undiagnosed chronic conditions, to guide early intervention efforts.
